# Effects of Glucose Oxidase Supplementation on the Growth Performance, Antioxidative and Inflammatory Status, Gut Function, and Microbiota Composition of Broilers Fed Moldy Corn

**DOI:** 10.3389/fphys.2021.646393

**Published:** 2021-06-16

**Authors:** Wenhui Qu, Jiaguo Liu

**Affiliations:** College of Veterinary Medicine, Nanjing Agricultural University, Nanjing, China

**Keywords:** glucose oxidase, inflammation, immunity, antioxidant ability, intestinal morphology

## Abstract

**Background:**

Glucose oxidase is widely used as a livestock feed additive owing to its beneficial effects on growth performance and antioxidant activity. However, little is known about the effects of the enzyme on intestinal health.

**Methods:**

To investigate the effects of glucose oxidase supplementation on the growth performance, intestinal function, and microbiota composition of broilers fed moldy corn, newly hatched Arbor Acres broilers were each randomly assigned to one of four groups, which were fed a basal diet (CON), a contaminated diet (10% moldy corn) (MC), a basal diet supplemented with 0.01% glucose oxidase (GOD), or a contaminated diet supplemented with 0.01% glucose oxidase (MCG).

**Results:**

We found that the average weight gain (ADG) of the MC group was significantly lower than those of the CON and GOD groups, and there were no significant differences in ADG between the MCG group and the CON and GOD groups. Intestinal morphology results revealed irregularly arranged villi and microvilli in the ilea from the MC group, whereas those from the other three groups were aligned regularly. Tight-junction protein analysis showed that both ZO-1 expression and claudin-4 expression in the MC group were significantly lower than those in the other groups. Inflammation cytokines analysis showed lower serum concentration of interleukin-10, as well as its mRNA expression in the ileum of the MC group, when compared with those of the other groups. Additionally, we observed lower glutathione peroxidase and total superoxide dismutase activity and higher malonaldehyde concentration in the MC group than those in the MCG group. The α and β diversity of microbiota profiling indicated that the cecal microbiota in the MC group differed from those in the other three groups.

**Conclusion:**

The results indicated that glucose oxidase supplementation was able to prevent the adverse effects from mycotoxin exposure on growth performance, antioxidant activity, inflammatory response, intestinal function, and microbiota composition in broilers. We suggested that glucose oxidase supplementation can be used in broilers to mitigate the adverse effects of moldy feed, and its benefits are due to its effect on intestinal microbiota composition.

## Introduction

The contamination of corn with mycotoxins is a major problem for livestock production worldwide, and especially in China (Wang et al., 2006). Some mycotoxins (e.g., aflatoxin B1, zearalenone, and deoxynivalenol) have been reported to have negative effects on chickens (e.g., diarrhea and reduced growth rate) (Beasley et al., 1980). Moreover, chronic consumption of low levels of moldy corn has been reported to increase the susceptibility of chickens to inflammatory and immune-related diseases (Afzal and Zahid, 2004). For these reasons, several studies have investigated the effectiveness of dietary adsorbents (Wan et al., 2013) and other additives (e.g., immune enhancers, antioxidants, and anti-inflammatory compounds) (Afzal and Zahid, 2004) in preventing mycotoxicity.

Glucose oxidase is a naturally occurring enzyme that is widely used as a livestock feed additive and has been reported to improve growth, immune function, antioxidant activity, and intestinal health (Chen et al., 2015; Cui et al., 2015; Wang et al., 2018). The enzyme also exerts antibacterial and antifungal effects by catalyzing the oxidation of β-D-glucose into 2-δ-gluconolactone and hydrogen peroxide (Wong et al., 2008) and is both non-toxic and low-residue (Wang et al., 2018). Furthermore, recent studies have reported that GO supplementation improves intestinal barrier function and cecal microbiota composition in broiler chickens (Wang et al., 2018; Wu et al., 2019). Therefore, the aim of the present study was to investigate whether providing glucose oxidase supplementation to broilers fed moldy corn could mitigate the negative effects of mycotoxins on growth performance, antioxidant activity, inflammatory response, immune function, intestinal function, and microbiota composition.

## Materials and Methods

### Experimental Design and Conditions

A total of 1,120 newly hatched male Arbor Acres broilers (7 replicates, 40 birds per replicate) were randomly assigned to one of four dietary treatment groups: the control (CON) group, fed a basal diet; the moldy corn (MC) group, fed a basal diet including 10% moldy corn; the GOD group, fed a basal diet supplemented with 0.01% glucose oxidase (1,000 U/g); or the moldy corn and glucose oxidase (MCG) group, fed a basal diet including 10% moldy corn and supplemented with 0.01% glucose oxidase. Glucose oxidase was purchased from CJ Youtell (Shanghai) Biotech Co., Ltd. Mash and water were provided *ad libitum* throughout the 42-day study. Environmental temperature was maintained at 32–34°C for the first 3 days, decreased by 3–4°C per week over the next 3 weeks, and then maintained at 22–24°C for the remainder of the experiment. The chicks were exposed to light every other 12 h. The basal diet was formulated according to recommendations from the National Research Council (1994) (Table 1). All procedures were reviewed and approved by the Institutional Animal Care and Use Committee of Nanjing Agricultural University (IACUC20200410).

**TABLE 1:** Composition and analyzed nutrient level of the basal diet (% as fed basis).

**Items**	**Composition,%**		**Items**	**Nutrient level,%**	
	**1–21 days**	**22–42 days**		**1–21 days**	**22–42 days**
Corn	57.67	59.80	ME (Mcal/kg)	3.72	3.08
Soybean meal	28.30	25.65	CP	20.51	19.28
Extruded soybean	8.00	8.00	Calcium	1.01	0.90
Soybean oil	1.90	3.00	Total phosphorus	0.66	0.63
Mountain flour	1.30	1.10	Non-phytate phosphorus	0.43	0.41
Calcium hydrophosphate	1.70	1.60	Methionine + cysteine	0.91	0.76
Sodium chloride	0.30	0.30	Methionine	0.56	0.42
Lysine, 98.5%	0.10	–	Lysine	1.15	1.01
DL-methionine	0.25	0.12	Tryptophan	0.24	0.22
Threonine	0.05	–	Threonine	0.81	0.72
Premix*	0.33	0.33			
Choline chloride	0.10	0.10			
Total	100	100			

*Supplied per kg of diet: Mn 59.4 mg, Zn 96.6 mg, Fe 66 mg, Cu 15 mg, Se 0.41 mg, I 0.38 mg, Va 8 000 IU, Vd 2 000 IU, Vk 2.0 mg, VB1 6.0 mg, VB3 6.0 mg, VB6 3.0 mg, VB5 14.0 mg, VE 30 IU, nicotinic acid 80 mg, and folic acid 1.5 mg. ME, metabolic energy; CP, crude protein.

### Mycotoxin Measurement

Dietary mycotoxin content was measured using commercially available enzyme linked immunosorbent assay test kits for aflatoxin B1, zearalenone, and deoxynivalenol (Meimian, Yancheng, China).

### Growth Performance

Broiler chicken weight (at 0, 21, and 42 days) and feed intake were recorded and used to calculate average daily weight gain (ADG), average daily feed intake (ADFI), and ratio of feed to gain (F/G) from 0 to 21 days, from 21 to 42 days, and from 0 to 42 days.

### Sample Collection

After 42 days, two broilers per replicate, excluding significant outliers in body weight, were randomly chosen for sampling. Blood samples for serum analysis were taken from the wing vein of each chick, after which the chicks were electrically stunned, exsanguinated, and scalded to permit intestinal sample collection. Serum was collected after the blood samples were centrifuged at 2,500 *g* at 4°C for 10 min and stored at –80°C for further analysis. Jejunal and ileal samples were taken and fixed with 4% formaldehyde for morphological observation, and the samples were also snap-frozen in liquid nitrogen and stored at –80°C until analysis of gene and protein expression. Samples of cecal content were collected for microbiota profiling.

### Serum Biochemical and Enzyme Analysis

Commercial assay kits (Meimian, Yancheng, China) were used to measure serum biochemical parameters, including the activities of several serum enzymes. Markers evaluated included alanine aminotransferase (ALT), aspartate transaminase (AST), glutathione peroxidase (GSH-PX), total superoxide dismutase (T-SOD), malonaldehyde (MDA), diamine oxidase (DAO), interleukin-10 (IL-10), transforming growth factor β (TGF-β), and secretory immunoglobulin A (sIgA).

### Total RNA Extraction and Quantitative Real-Time Polymerase Chain Reaction

Total RNA was extracted using TRIzol reagent and treated with DNase. Then, reverse transcription was performed using the PrimeScript reverse transcription reagent kit (TIANGEN, Beijing, China). Quantitative real-time polymerase chain reaction was performed as described previously (He et al., 2020), using a Light Cycler 480 system (Roche, Shanghai, China), specific primers (Table 2), and SYBR Green mix (TIANGEN). The comparative *C*_*t*_ value method was used to quantify mRNA expression relative to GAPDH expression.

**TABLE 2:** Primers for reverse transcriptase–quantitative polymerase chain reaction.

**Target gene**	**Primer sequence (5′–3′)**
*Gpx1*	F: TGCGCCCGATGTTTTCAAAG R: AACGTTACCCAGACTCACGG
*Sod1*	F: CACGGTGGACCAAAAGATGC R: GATGCAGTGTGGTCCGGTAA
*Sod2*	F: TACAGCTCAGGTGTCGCTTC R: GCGAAGGAACCAAAGTCACG
*IL-10*	F: CAGACCAGCACCAGTCATCA R: TCCCGTTCTCATCCATCTTCTC
*TGF-*β*1*	F: TCCAATATGGTGGTCCGTGC R: ACCCCCAAAAAGGGAACCATC
*GAPDH*	F: TGCTGCCCAGAACATCATCC R: ACGGCAGGTCAGGTCAACAA

*Gpx1, glutathione peroxidase 1; Sod, superoxide dismutase; IL-10, interleukin-10; TGF-β1, transforming growth factor β.*

### Intestinal Morphology

The samples were fixed using formalin, embedded in paraffin, sliced into sections, and stained using hematoxylin and eosin, and then observed using a light microscope, as described previously (Zhou et al., 2018). For ultrastructure observation, the samples were sequentially fixed using glutaraldehyde and then osmium tetroxide, dehydrated, embedded, sliced into ultrathin sections, stained using uranyl acetate and lead citrate, and observed using a transmission electron microscope (Wang et al., 2020).

### Western Blot Analysis

Proteins were extracted; separated using sodium dodecyl sulfate–polyacrylamide gel electrophoresis; blotted onto a nitrocellulose membrane; incubated overnight at 4°C with primary antibodies against ZO-1, claudin-4, nuclear factor κB (NF-κB) p65, and phospho–NF-κB p65 (pNF-κB) (Bioss, Beijing, China); incubated again with related secondary antibodies; and analyzed as described previously (Zhou et al., 2017a). Densitometric results were analyzed with ImageJ software.

### Gut Microbiota Profiling

Total DNA was extracted from each of the cecal content samples, and specific primers were used to amplify microbial barcode markers (16S rRNA gene, V3–V4 region). Sequencing libraries were generated and analyzed as described previously (Li et al., 2020; Panaite et al., 2020), and operational taxonomic units were determined using Tax4Fun software.

### Statistical Analysis

Data were analyzed according to a 2 × 2 factorial design using a mixed procedure (PROC MIXED) in SAS version 9.2 (SAS Institute, Cary, NC, United States). Replicate groups were designated as the experimental units for the analysis of growth performance, whereas individual birds were designated as the experimental units for the analysis of the other parameters. The statistical model evaluated the effects of moldy corn, glucose oxidase, and their interaction. Mean values were considered significantly different at *P* ≤ 0.05. Data were expressed as mean ± standard error.

## Results

### Broiler Growth Performance

The moldy corn contained 54.1 μg/kg aflatoxin B1, 7.5 mg/kg zearalenone, and 2.01 mg/kg deoxynivalenol, whereas uncontaminated corn contained only 0.04 mg/kg deoxynivalenol and negligible levels of aflatoxin B1 and zearalenone.

Overall, no interactions between moldy corn and glucose oxidase were observed for ADG, nor was there a significant difference between the ADG values of the CON and GOD groups. For the first 21 days, the ADG of the MC group was significantly lower than those of the other three treatment groups, and the ADG of the MCG group was significantly lower than that of the GOD group (Table 3). Meanwhile, for both the second half of the experiment (21–42 days) and the overall experimental period (0–42 days), the ADG of the MC group was significantly lower than those of the CON and GOD groups, and there were no significant differences between the ADG of the MCG group and those of the other three treatment groups. However, no significant difference in the ADG between the CON and MCG group was observed during the overall experimental period. In addition, neither moldy corn nor glucose oxidase significantly affected ADFI or F/G, and no interactions between moldy corn and glucose oxidase were observed for either parameter.

**TABLE 3:** Growth performance of chickens.

	**CON**	**MC**	**GOD**	**MCG**	**SEM**	***P*-value**		
						**GOD**	**MC**	**G × M**
**From days 0–21**								
ADG, g/d	28.11^ab^	26.63^c^	28.75^a^	27.73^b^	0.246	0.002	<0.001	0.368
ADFI, g/d	41.35	40.21	41.16	40.59	0.322	0.771	0.064	0.281
F/G	1.47	1.51	1.43	1.46	0.020	0.054	0.096	0.892
**From days 22–42**								
ADG, g/d	76.17^a^	70.36^b^	77.03^a^	74.72^ab^	1.473	0.090	0.011	0.245
ADFI, g/d	142.80	137.46	141.76	141.21	2.021	0.510	0.158	0.247
F/G	1.88	1.96	1.85	1.90	0.046	0.317	0.191	0.798
**From days 0–42**								
ADG, g/d	52.14^a^	48.50^b^	52.89^a^	51.23^ab^	0.737	0.027	0.001	0.192
ADFI, g/d	92.08	88.83	91.46	90.90	1.042	0.495	0.081	0.210
F/G	1.77	1.83	1.73	1.78	0.032	0.164	0.104	0.788

*^*a,b,c*^Means within a row not sharing a common superscript differ significantly P ≤ 0.05. n = 7.*

*ADG, average daily weight gain; ADFI, average daily feed intake; F/G, ratio of feed to gain. CON, broilers fed a basal diet; MC, broilers fed a basal diet including 10% moldy corn; GOD, broilers fed a basal diet supplemented with 0.01% glucose oxidase; MCG, broilers fed a basal diet including 10% moldy corn and supplemented with 0.01% glucose oxidase.*

### Intestinal Morphology, Permeability, and Function

**FIGURE 1:**
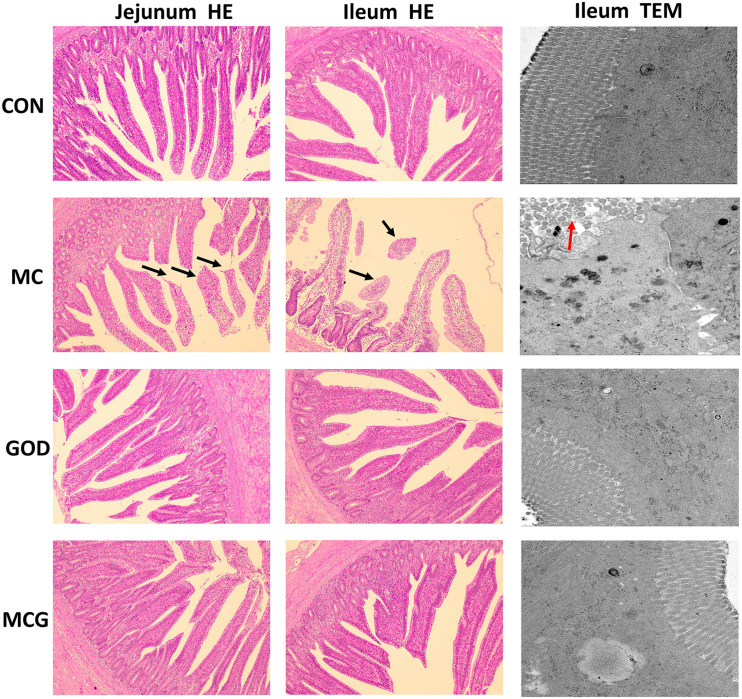
Effects of glucose oxidase on intestinal morphology in broiler chickens fed mold contaminated corn. Results of jejunum (left) and ileum morphology (middle) by HE staining (magnification × 100). Results of ileum morphology (right) by transmission electron microscope (magnification × 5,000). Black arrows, partial loss of villi; red arrows, irregularly arranged microvilli.

**FIGURE 2:**
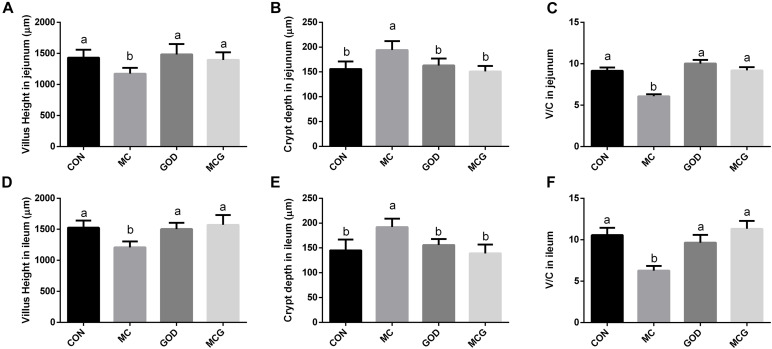
Effects of glucose oxidase on villus height and crypt depth in broiler chickens fed mold contaminated corn. **(A)** Villus height in jejunum; **(B)** crypt depth in jejunum; **(C)** V/C in jejunum; **(D)** Villus height in ileum; **(E)** crypt depth in ileum; **(F)** V/C in ileum. V/C, the ratio of villus height to crypt depth. ^*a,b*^Means with different letters within columns indicate significant differences at *P* ≤ 0.05. *n* = 3.

Hematoxylin–eosin staining revealed partial atrophy of jejunal and ileal villi in the MC group, but not in the other groups (Figure 1). Villus height and the ratio of villus height to crypt depth in both jejunum and ileum were significantly higher, whereas crypt depth was significantly lower in the MC group than those in the other three groups. No significant changes of the aforementioned parameters were observed among the CON, GOD, and MCG groups (Figure 2). Transmission electron microscopy revealed irregularly arranged microvilli in the ilea from the MC group, whereas those from the other three groups were aligned regularly.

**FIGURE 3:**
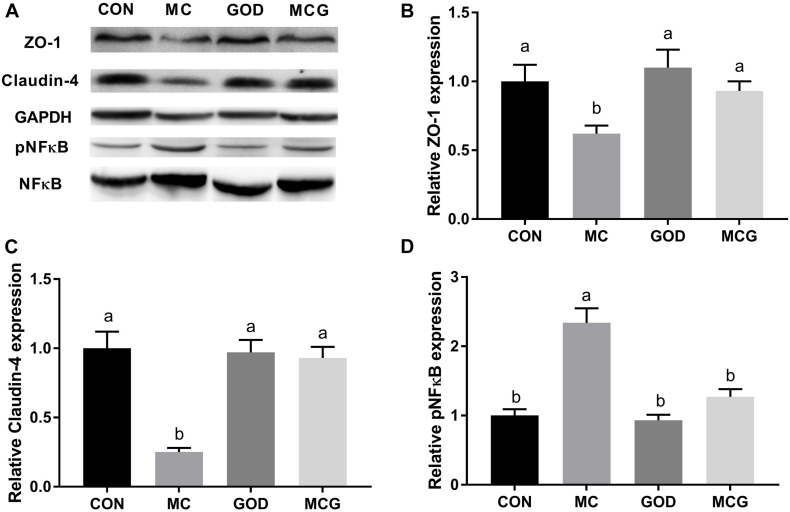
Effects of glucose oxidase on intestinal protein expression of ZO-1, claudin-4, and NF-κB in the broiler chickens fed mold contaminated corn. **(A)** Western blotting results; relative protein expression of ZO-1 **(B)**, claudin-4 **(C)**, and phosphorylated NF-κB **(D)**. ZO-1 and claudin were normalized to GAPDH. pNF-κB was normalized to NF-κB. ^*a,b*^Means with different letters within columns indicates significant differences at *P* ≤ 0.05. *n* = 3.

Significant interactions between moldy corn and glucose oxidase were observed for serum DAO level, with DAO level in the MCG group being significantly higher than in the MC group, whereas there was no significant difference between the GOD and CON groups (Table 4). In contrast, no interactions between moldy corn and glucose oxidase were observed for the expression of ZO-1 or claudin-4 in ileum tissue, and there was no significant difference between ZO-1 or claudin-4 levels in the CON and GOD groups. However, both ZO-1 and claudin-4 levels in the MC group were significantly lower than those in the other groups (Figures 3A–C). No significant changes of the abovementioned parameters were observed among the CON, GOD, and MCG groups.

**TABLE 4:** Serum biochemical and enzyme analysis.

	**CON**	**MC**	**GOD**	**MCG**	**SEM**	***P*-value**		
						**GOD**	**MC**	**G × M**
**Antioxidative status**								
GSH-PX, μmol/mL	8.20^ab^	3.53^c^	8.39^a^	7.42^b^	0.209	<0.001	<0.001	<0.001
T-SOD, U/mL	49.0^a^	28.2^b^	48.8^a^	42.8^a^	1.863	0.001	<0.001	0.001
MDA, nmol/mL	4.34^b^	8.62^a^	4.17^b^	5.07^b^	0.247	<0.001	<0.001	<0.001
**Immune and inflammatory status**								
sIgA, μg/mL	1.43^a^	0.96^b^	1.46^a^	1.34^a^	0.040	<0.001	<0.001	<0.001
IL-10, ng/mL	6.20^a^	2.82^b^	6.05^a^	5.67^a^	0.149	<0.001	<0.001	<0.001
TGF-β, ng/mL	59.9^b^	84.1^a^	55.0^b^	63.2^b^	2.196	<0.001	<0.001	0.001
**Liver function**								
AST, U/mL	79.2^b^	99.9^a^	83.6^b^	83.0^b^	2.64	0.027	0.001	<0.001
ALT, U/mL	19.3^b^	25.1^a^	18.7^b^	20.0^b^	0.641	<0.001	<0.001	0.001
**Intestinal function**								
DAO, U/mL	11.8^b^	21.2^a^	11.6^b^	13.4^b^	0.616	<0.001	<0.001	<0.001

*^*a,b,c*^Means within a row not sharing a common superscript differ significantly P ≤ 0.05. n = 7.*

*ALT, alanine aminotransferase; AST, aspartate transaminase; GSH-PX, glutathione peroxidase; T-SOD, total superoxide dismutase; MDA, malonaldehyde; DAO, diamine oxidase; IL-10, interleukin-10; TGF-β, transforming growth factor β; sIgA, secretory immunoglobulin A. CON, broilers fed a basal diet; MC, broilers fed a basal diet including 10% moldy corn; GOD, broilers fed a basal diet supplemented with 0.01% glucose oxidase; MCG, broilers fed a basal diet including 10% moldy corn and supplemented with 0.01% glucose oxidase.*

### Inflammatory Response, Immune Function, and Antioxidant Activity

The ileal pNF-κB level was significantly higher in the MC group than in the other three groups (Figure 3), and serum concentrations of IL-10 and TGF-β, as well as levels of IL-10 and TGF-β mRNA expression in the ileum, differed significantly between the MC group and the other groups (Figure 3 and Table 4). Moreover, the serum sIgA concentration in the MC group was significantly lower than in the other three groups, whereas serum concentrations of AST and ALT were significantly higher (Table 4). No significant changes of the abovementioned parameters were observed among the CON, GOD, and MCG groups.

**FIGURE 4:**
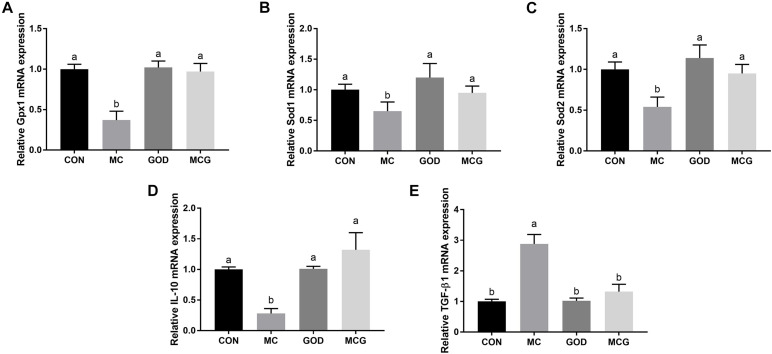
Effects of glucose oxidase on intestinal mRNA expression of antioxidative stress- and inflammation-associated genes in the broiler chickens fed mold contaminated corn. Relative expression of *Gpx1* **(A)**, *Sod1* **(B)**, *Sod2* **(C)**, *IL-10* **(D)**, and *TGF-*β*1* **(E)**. ^*a,b*^Means with different letters within columns indicates significant differences at *P* ≤ 0.05. *n* = 7.

Significant interactions between moldy corn and glucose oxidase were observed for serum GSH-PX activity, T-SOD activity, and MDA concentration; the MCG group showed higher GSH-PX and T-SOD activity and lower MDA concentration than did the MC group, but there was no such difference between the GOD and CON groups (Table 4). However, no interactions between moldy corn and glucose oxidase were observed with regard to the ileal expression of GSH-PX (*Gpx1*), superoxide dismutase (*Sod1*), or *Sod2*, nor was there a significant difference in the expression of these genes between the CON and GOD groups. However, the expression of Gpx1, Sod1, and Sod2 was significantly lower in the MC group than in the other groups (Figure 4). No significant changes of the aforementioned parameters were observed among the CON, GOD, and MCG groups.

### Cecal Microbiota

**FIGURE 5:**
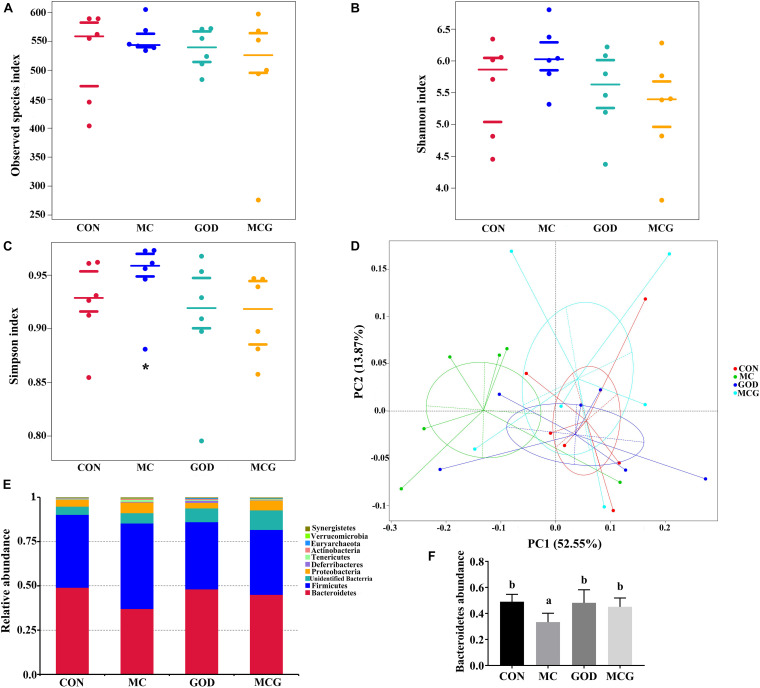
Effects of glucose oxidase on cecal microbiota composition in broiler chickens fed mold contaminated corn. **(A)** Observed species index; **(B)** Shannon index; **(C)** Simpson index; **(D)** PCoA plot of the microbiota based on an unweighted UniFrac metric; **(E)** relative abundance of predominant bacteria at the phylum level; **(F)** relative abundance of *Bacteroidetes*. *Significant differences at *P* ≤ 0.05. *n* = 7.

The α diversity of the cecal microbiota showed that the Simpson index values in the MC group was significantly higher than those in the other three groups, whereas there were no significant differences between groups with regard to the observed species or Shannon index values (Figure 5). Unweighted principal coordinates analysis indicated a clear difference in β-diversity in the MC group. Top-10 group analysis indicated that *Firmicutes*, *Bacteroidetes*, and *Proteobacteria* were the main phyla in the cecal community, with the abundance of *Bacteroidetes* taxa being significantly lower in the MC group than in the other three groups.

## Discussion

Glucose oxidase is used extensively in poultry feed production and has been reported to enhance growth rate and antioxidant activity (Tang et al., 2016; Wu et al., 2019; Zhang et al., 2020), as well as to exert other beneficial effects such as amelioration of mycotoxicity and improvement of both digestive function and intestinal microbiota composition (Cruz et al., 2012; Wu et al., 2019). In this study, even though glucose oxidase supplementation did not significantly increase broiler growth performance, it maintained ADG, reduced inflammatory response, and improved antioxidant activity and immune function in broilers exposed to mycotoxins. Glucose oxidase supplementation also protected the intestine from mycotoxicity by maintaining permeability, preventing villous disruption, and improving the expression of tight-junction proteins. These results suggest that supplementing broiler diets with glucose oxidase is useful for mitigating the adverse effects of mycotoxin consumption.

One of the most important and notable effects of glucose oxidase is its ability to improve the antioxidant activity or directly function as an antioxidant (Wong et al., 2008). Interestingly, in contrast to previous studies (Wang et al., 2018; Wu et al., 2019), this study found that glucose oxidase did not affect oxidative status in broilers fed an uncontaminated basal diet, possibly because the broilers used in the present study were managed under a relatively favorable environment and endured less stress. Indeed, it is possible that glucose oxidase only improves oxidative status when broilers are under stress; this is supported by our finding that glucose oxidase improved serum GSH-PX and T-SOD activity and intestinal Gpx1, Sod1, and Sod2 expression in broilers fed moldy corn.

The intestine is the most important immune organ, and sIgA, which is secreted by intestinal epithelial cells, plays a key role in immunity (Schmidt et al., 2007; Janardhana et al., 2009; Lammers et al., 2010). In the present study, moldy corn reduced sIgA levels, which suggested a disruption of immune function. However, sIgA levels were elevated in the GOD group, indicating a beneficial effect on immunity. Moreover, glucose oxidase supplementation reduced the inflammatory response caused by mycotoxin exposure. Importantly, the results of the present study also suggest that glucose oxidase exerts its effects by inhibiting the NF-κB signaling pathway. Thus, glucose oxidase appears to function as an immune modulator in the intestines of broilers.

The permeability of the intestinal barrier plays an important role in gut health (Gosain and Gamelli, 2005; Bischoff et al., 2014), and barrier function is correlated with antioxidant activity, inflammatory response, and immune function in the intestine (Zhou et al., 2017b; Wang et al., 2018; Fan et al., 2020). In the present study, glucose oxidase supplementation maintained the shape and regularity of intestinal villi and reduced DAO levels in broilers fed moldy corn, thereby demonstrating beneficial effects on intestinal integrity. Tight junctions are a vital component of the intestinal barrier (Kucharzik et al., 2001), and in the present study, intestinal expression of the tight-junction proteins ZO-1 and claudin-4 was significantly increased by glucose oxidase supplementation. Taken together, these results suggest that glucose oxidase protected the intestinal barriers of the broilers from damage caused by mycotoxins.

It has been suggested that glucose oxidase affects intestinal microbiota composition via its role in consuming oxygen and producing hydrogen peroxide and gluconic acid (Kapat et al., 1998). Wu et al. (2019) reported that dietary glucose oxidase could affect microbiota composition, specifically by increasing the abundance of *Ruminococcaceae* and *Firmicutes*. However, no significant changes in microbiota composition between control broilers and broilers only supplemented with glucose oxidase were observed in the present study, possibly because different levels of glucose oxidase were added to the different experimental diets. Nevertheless, both α and β diversity indices suggested that glucose oxidase maintained an intestinal microbiota composition that would otherwise have been disrupted by exposure to mycotoxins. Furthermore, glucose oxidase supplementation has been observed to increase the relative abundance of *Bacteroidetes* taxa, which is usually correlated with propionate content (Salonen et al., 2014), as *Bacteroidetes* produce propionic acid during the degradation of dietary carbohydrates (Louis and Flint, 2017). This is especially interesting because propionic acid is an efficient fungistat, and dietary supplementation with propionic acid has been reported to restore the nutritional value of diets containing moldy corn (Bartov, 1983). Consequently, the beneficial effects of glucose oxidase on mycotoxin-exposed broilers could be due to its influence on the relative abundance of *Bacteroidetes* taxa. However, further investigations are needed to elucidate the mechanism by which glucose oxidase might influence the abundance of *Bacteroidetes* taxa and to confirm whether increased abundance of *Bacteroidetes* taxa promotes the production of propionic acid in the intestines of mycotoxin-exposed broilers.

In conclusion, we found that glucose oxidase supplementation had a protective effect on growth performance, inflammatory response, antioxidant activity, and immune function in broilers fed diets containing moldy corn. Moreover, glucose oxidase protected the intestine from damage caused by mycotoxins, preventing villous disruption and maintaining permeability and barrier function. Glucose oxidase also improved intestinal microbiota composition in mycotoxin-exposed broilers, which suggests that the beneficial effects of glucose oxidase may be mediated by the microbiota.

## Data Availability Statement

The raw data supporting the conclusions of this article will be made available by the authors, without undue reservation.

## Ethics Statement

The animal study was reviewed and approved by the Institutional Animal Care and Use Committee of Nanjing Agricultural University.

## Author Contributions

WQ and JL designed the experiment and drafted the manuscript. WQ carried out the animal trials and sample analysis, and did data analysis work. JL was responsible for the integrity of the work as a whole. Both the authors reviewed and approved the final manuscript.

## Conflict of Interest

The authors declare that the research was conducted in the absence of any commercial or financial relationships that could be construed as a potential conflict of interest.
